# Supplementary material for: Sonographic, Demographic, and Clinical Characteristics of Pre- and Postmenopausal Women with Endometrial Cancer; Results from a Post Hoc Analysis of the IETA4 (International Endometrial Tumor Analysis) Multicenter Cohort
Source: Diagnostics (Basel). 2023 Dec 19;14(1):1. doi: 10.3390/diagnostics14010001 (PMC10802150; doi:10.3390/diagnostics14010001)
Supplement: Supplementary file 1 [file diagnostics-14-00001-s001.zip › diagnostics-2735367-supplementary.pdf]

Table S1. Significance testing by the Benjamini-Yakutieli procedure for each hypothesis test.

Comparison	Unadjusted *p*	Benjamini-Yakutieli significance
Duration of abnormal bleeding	0.0009	significant
Endometrial midline	0.0009	significant
FIGO surgical stage	0.0009	significant
Histologic type	0.0009	significant
Parity	0.0009	significant
Regular EM-junction	0.0009	significant
Risk group	0.0009	significant
Regular EM-junction, in relation to risk group, pre-	0.0009	significant
Defined tumor, in relation to risk group, post-	0.0009	significant
BMI, in relation to risk group, post-	0.0009	significant
Regular EM-junction, in relation to risk group, post-	0.0009	significant
Endometrial echogenicity, in relation to risk group, post-	0.0009	significant
Color score, in relation to risk group, post-	0.0009	significant
Vascular pattern, in relation to risk group, post-	0.0009	significant
Waist group, in relation to risk group, post-	0.001	significant
Adenomyosis suspected	0.001	significant
Defined tumor	0.001	significant
Family Hx colon cancer	0.001	significant
Grade among endometrioid	0.0012	significant
Waist circumference	0.002	significant
**Endometrial thickness by waist group, post-MP**	0.005	not significant
**Bright edge sign**	0.007	not significant
**Body constitution**	0.024	not significant
**Endometrial thickness by BMI, post-MP**	0.036	not significant
Fibroids present	0.099	not significant
Endometrial thickness	0.1315	not significant
Color score, in relation to risk group, pre-	0.156	not significant
Alcohol use	0.193	not significant
Defined tumor, in relation to risk group, pre-	0.196	not significant
Vascular pattern, in relation to risk group, pre-	0.216	not significant
Endometrial thickness by waist, pre-MP	0.219	not significant
Endometrial echogenicity, in relation to risk group, pre-	0.232	not significant
Excersice	0.243	not significant
BMI	0.2899	not significant
Family Hx endometrial cancer	0.311	not significant
Measurable endometrium	0.356	not significant
Endometrial echogenicity	0.387	not significant
Smoking	0.398	not significant
Abnormal bleeding	0.428	not significant
Endometrial midline, in relation to risk group, post-	0.431	not significant
Vascular pattern	0.439	not significant
Color score	0.545	not significant
Endometrial thickness by BMI, pre-MP	0.6052	not significant
Weight	0.671	not significant
BMI, in relation to risk group, pre-	0.696	not significant
Family Hx other cancer	0.697	not significant
Family Hx ovarian cancer	0.717	not significant
Tumor volume	0.786	not significant
Endometrial midline, in relation to risk group, pre-	0.818	not significant
Bra cup size	0.87	not significant
Family Hx breast cancer	0.893	not significant
Hormonal treatment	1	not significant
Waist group, in relation to risk group, pre-	1	not significant

Bold typeface indicates change in statistical significance from the uncorrected overall critical *p*-value of 0.05 to the corrected *p*-value of 0.0041
